# From observation to participation: a needs assessment of clinical clerkship in Korean medicine toward competency-based education

**DOI:** 10.3389/fmed.2026.1969260

**Published:** 2026-10-14

**Authors:** Seung A. Jeong, JongHyeon Hong, Sang Yun Han, Eunbyul Cho

**Affiliations:** 1 College of Korean Medicine, Wonkwang University, Iksan, Republic of Korea; 2 Korean Medicine Hospital, Wonkwang University, Iksan, Republic of Korea; 3 Department of Korean Medicine Education, College of Korean Medicine, Wonkwang University, Iksan, Republic of Korea; 4 Research Center of Traditional Korean Medicine, Wonkwang University, Iksan, Republic of Korea; 5 Department of Diagnostics, College of Korean Medicine, Wonkwang University, Iksan, Republic of Korea

**Keywords:** clinical clerkship, competency-based education, Korean medicine, mixed-methods study, needs assessment, traditional medicine

## Abstract

**Background:**

Health professions education is shifting from simple observation to competency-based education, and Korean Medicine is no exception. Clerkship is increasingly regarded as the stage at which students acquire the competencies needed to deliver primary care independently. However, what learners experience and require in clerkship remains largely unexamined in traditional medicine. This study identified the current state of clerkship education and learner needs at a college of Korean Medicine and explored the causes and solutions to the problems it uncovered.

**Methods:**

We used an explanatory mixed-methods design with partially overlapping data collection, grounded in pragmatism. Forty-two fourth-year students who had completed a clerkship at the College of Korean Medicine, Wonkwang University, completed a survey in 2025; seven survey respondents also participated in in-depth interviews. Preliminary survey responses informed the interview guide, and the qualitative findings were used to explain and contextualize the quantitative results. Paired *t*-tests were used to examine importance–performance gaps across five competency domains (history taking, physical examination and diagnostic testing, communication skills, clinical reasoning and treatment planning, and clinical skills). Priorities were derived using the Borich needs assessment and the Locus for Focus model. Interview data underwent thematic analysis structured according to educational content, methods, and environment.

**Results:**

Most respondents (83.3%) were satisfied with clerkship education, yet 42.9% considered change necessary, most often citing “variation in clerkship across departments.” Performance fell below the importance level in all five domains. Borich priorities ranked communication skills the first, followed by clinical skills. The Locus for Focus model placed physical examination and diagnostic testing in the highest-priority quadrant. The interviews identified four issues: limited participation in patient care, the absence of a systematic communication curriculum, too few opportunities for repeated skills practice, and variations in clerkship delivery across hospitals and departments.

**Conclusions:**

Clerkship education in colleges of Korean Medicine may benefit from moving beyond passive observation toward structured participation and competency-focused training centered on communication and clinical skills. These findings offer an empirical basis for identifying priority areas in clerkship redesign and serve as foundational evidence for informing future competency-based clerkship development in traditional medicine.

## 1 Introduction

In medical education, clinical clerkship plays a pivotal role that extends beyond knowledge transmission, cultivating practitioners equipped with practical primary care competencies (1, 2). Medical education has recently shifted from an observation-centered approach, in which learners passively watch the care process, to competency-based education (CBE), in which learners progressively participate in actual patient care under faculty supervision. CBE has become a key criterion for determining the quality of clinical clerkships (3). CBE specifies the abilities a graduate must be able to demonstrate, sequential training around their progressive attainment, and anchor assessment in observed performance rather than in the time served (4). For example, in Germany, four years of clinical clerkship is designed to ensure that students can provide a high standard of patient care regardless of the training site. In the United States, clerkship similarly guarantees opportunities for direct patient interaction so that students can acquire the communication skills required of a physician (5).

In East Asian traditional medicine as well, clinical clerkship has increasingly been emphasized as a core step for implementing CBE (6). Korea has 12 colleges of Korean Medicine, where students become eligible for the national licensing examination after completing a six-year undergraduate curriculum, a seven-year integrated bachelor's–master's curriculum, or a four-year graduate-entry master's program (7). Unlike most medical school graduates, who undergo postgraduate training as interns and residents, only approximately 13% of licensed Korean Medicine doctors held specialist qualifications as of 2023, and the majority provide primary care (8). Undergraduate clerkship is therefore the last structured opportunity to equip students with the competencies required to deliver primary care independently from the day they graduate. Recognizing this, the Institute of Korean Medicine Education and Evaluation, the accrediting body for colleges of Korean Medicine, used its 2022 accreditation standards to mandate strengthened clerkship: expanded clerkship hours, compulsory objective structured clinical examinations (OSCEs) and clinical performance examinations (CPXs), and greater active student participation in patient care (9). Despite these institutional changes, however, what learners actually experience and require in real clerkship settings has not yet been sufficiently explored.

While prior studies have explored satisfaction and potential improvements in Korean Medicine clerkship education, there is a lack of in-depth examination of the current state of education and necessary enhancements from the learner's perspective. A survey of 20 clinical faculty members on the operation of the clerkship curriculum and its improvement needs identified the development of diverse assessment methods and the expansion of training facilities as the highest priorities; however, it was confined to the faculty's perspective and did not address the difficulties learners themselves encounter (10). Another study, examining the determinants of clerkship satisfaction among third- and fourth-year Korean Medicine students (11), found that enthusiastic faculty guidance and sufficient opportunities for patient interaction and hands-on skills practice shaped satisfaction. However, since it relied on closed survey items alone, it could not establish which specific factors depressed satisfaction. A recent study introduced workplace-based learning into the acupuncture and moxibustion clerkship at Wonkwang University. Through interviews with graduates three years after their licensure revealed the challenges that newly licensed Korean Medicine doctors face immediately after graduation, as well as their unmet needs related to their clerkship experience. Nevertheless, the study did not derive improvements for the overall clerkship curriculum (12).

Learners are both direct beneficiaries of clerkships and key stakeholders in their improvement. Accurately identifying their needs is the starting point for redesigning. Therefore, this study aimed to characterize the current state of clerkship education and learner needs at a college of Korean Medicine, and to explore the causes of the largest performance gaps together with potential solutions. We treated needs assessment as an initial diagnostic step for identifying local priorities for clerkship improvement. The findings were subsequently considered in relation to the potential future development of competency-based clinical education. Accordingly, we applied a needs-assessment methodology that derives priorities from the gap between the desired and current state, combining the Borich needs assessment model, which weights the importance–performance discrepancy by importance, with the Locus for Focus model, which plots the discrepancy in quadrants for intuitive prioritization, which together account for both statistical significance and practical importance. Since quantitative needs analysis alone cannot capture the context in which problems arise or the specific difficulties learners encounter, we adopted an explanatory mixed-methods design with partially integrated survey and interview data (13).

Placing this work within international CBE literature also matters. Works such as entrustable professional activities (EPAs), milestones, and programmatic and workplace-based assessments have been developed largely within biomedical curricula (14–16), and evidence on how they translate to traditional medicine education remains scarce. Korean Medicine offers an instructive case; most graduates enter primary care directly without an intervening residency, and its diagnostic reasoning rests on pattern identification, a process with no direct counterpart in biomedicine. Establishing what learners in this setting judge important and where they fall short is a precondition for adapting CBE frameworks.

The present study aimed to provide foundational data for designing clerkship education programs that reflect learners’ needs. The findings can inform evidence-based redesign of the priority areas identified here, the establishment of standardized assessment and feedback systems, and future deliberations by policy bodies, such as the Institute of Korean Medicine Education and Evaluation, on revising accreditation standards and the framework for clinical competency education in Korean Medicine (6). More broadly, this study may suggest a wider conversation about implementing CBE in traditional medicine.

## 2 Methods

Clinical clerkship at the College of Korean Medicine, Wonkwang University, the setting of this study, comprises three components: a 24-week department-based clinical rotation across three affiliated hospitals, incorporating CPX for major clinical presentations and OSCE for clinical skills; an 8-week program held once weekly at the clinical skills center, in which students are divided into patient and doctor groups for role-play on clinical presentations (17). The patient group develops a scenario independently while the doctor group prepares clinical reasoning, a diagnosis, and a treatment plan; and a 2-week community-based clerkship at the college's affiliated clinics, chosen freely by the student and followed by a written report (Table 1).

**Table 1:** Components of clinical clerkship at the College of Korean Medicine, Wonkwang University.

Category	Clinical rotation (24)			CPX role-playing (8)	Primary care clerkship (2)
**Venue**	A hospital (8)	B hospital (8)	C hospital (8)	Clinical Skills Center	Institution selected by student
**Department**	Cardiology and Neurology (2)	Cardiology and Neurology (1)	Cardiology and Neurology (1)	–	–
	Acupuncture and Moxibustion (1)	Acupuncture and Moxibustion (1)	Acupuncture and Moxibustion (1)		
	Korean Medicine Rehabilitation (1)	Korean Medicine Rehabilitation (1)	Korean Medicine Rehabilitation (1)		
	Korean Medicine Obstetrics and Gynecology (1)	Korean Medicine Obstetrics and Gynecology (1)	Korean Medicine Obstetrics and Gynecology (1)		
	Ophthalmology & otolaryngology & dermatology (1)	Pediatrics (1)	Ophthalmology & otolaryngology & dermatology (1)		
	Hepatology (1)	Gastroenterology (1)	Gastroenterology (1)		
	Neuropsychiatry (1)	Neuropsychiatry (1)	Pulmonology (1)		
		Sasang Constitutional Medicine (1)	Nephrology (1)		

Numbers in parentheses indicate the duration of each component in weeks.

### 2.1 Participants

The participants were fourth-year students at the College of Korean Medicine, Wonkwang University who had completed a clinical clerkship in 2025. Only students who voluntarily provided written informed consent to participate in the survey and interviews were included. Of the 91 eligible students, 42 completed the survey (response rate: 46.2%).

### 2.2 Study design

This study employed an explanatory mixed-methods design with partially overlapping data collection, grounded in a pragmatist perspective to support the practical improvement and redesign of the clerkship program at the College of Korean Medicine (18). Preliminary survey responses informed the interview guide and participant recruitment, whereas the complete quantitative dataset was analyzed after the survey closed. The qualitative findings were then used to explain and contextualize the quantitative results (Figure 1). This study was approved by the Institutional Review Board of Wonkwang University (WKIRB-202510-SB-087; approved on October 15, 2025), and all participants provided written informed consent.

**Figure 1:**
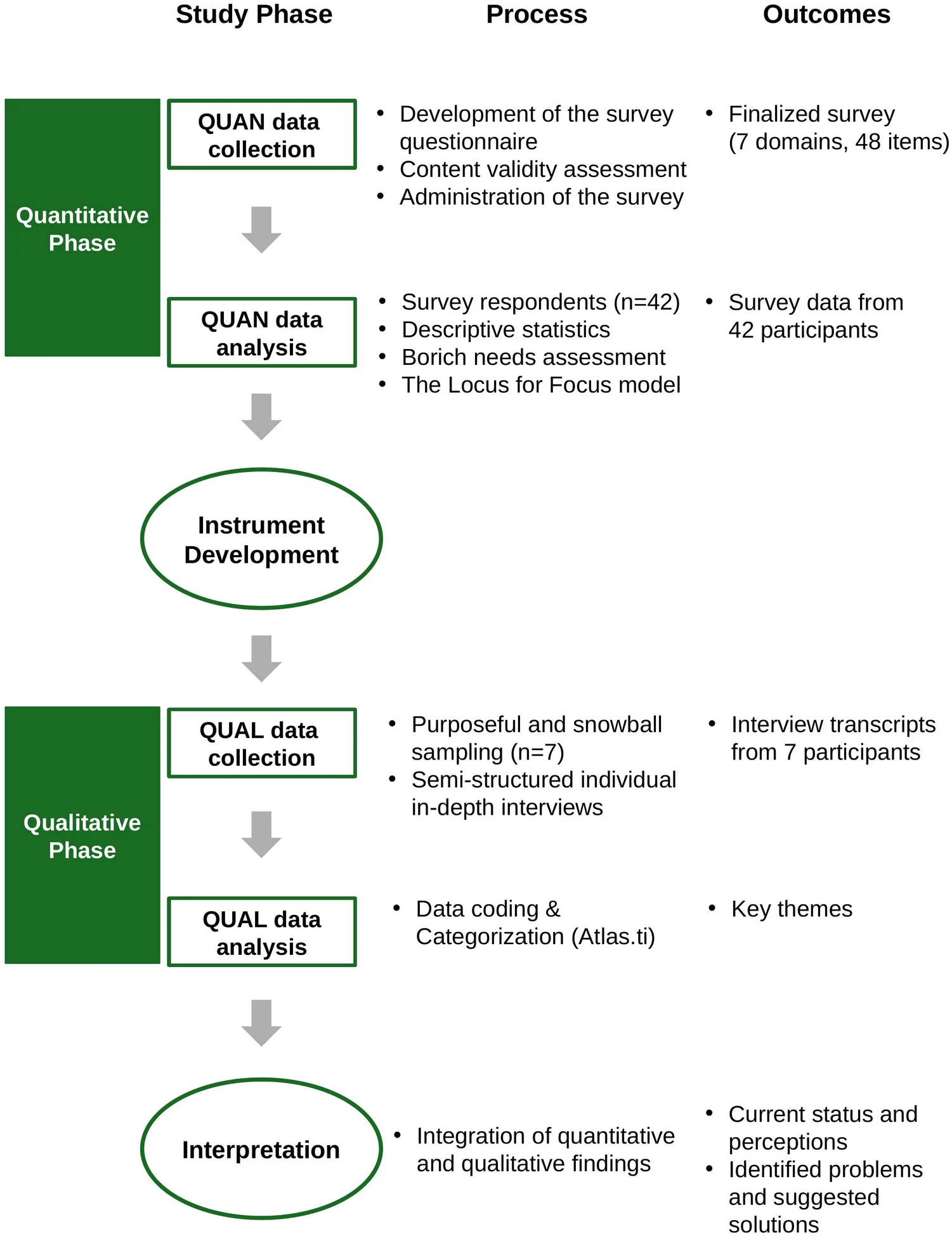
Flowchart of the explanatory mixed-methods design with partially overlapping data collection. The study proceeded in two phases. In the quantitative (QUAN) phase, survey data were collected and analyzed to assess educational needs and establish priorities, and a semi-structured interview guide was developed from those results. In the subsequent qualitative (QUAL) phase, in-depth interviews were conducted and analyzed to explain the quantitative findings. The two data collection periods partially overlapped, as interviewees were recruited on a rolling basis from students who had already completed the survey. Data from both phases were then integrated to identify current problems and propose solutions.

### 2.3 Survey

The survey items were developed to assess learners’ overall perceptions of their current clerkship education and to identify the causes of the problems they reported (19). Two researchers drafted the initial items based on prior studies of clinical clerkship education (10, 11); the items were then finalized following a content validity assessment by three clinician educators with more than five years of experience supervising clinical clerkship and three Doctors of Education. Content validity was rated on a 4-point scale (1 = not at all valid, 2 = not valid, 3 = valid, 4 = very valid), from which the item-level content validity index (I-CVI) was calculated. Items reaching an I-CVI of 0.83 or above—rated 3 or 4 by at least 5 of the 6 reviewers—were retained, while those falling below this threshold were revised in line with reviewer feedback.

The final survey comprised 41 multiple-choice items and 7 open-ended questions across seven domains: participant characteristics, pre-clerkship education, overall perception of clinical clerkship education, CPX and OSCE, clinical rotations, community-based clerkship, and future improvements in clinical clerkship. Importance and performance were rated on a 5-point Likert scale (Supplementary File 1). The five competency domains—history taking, physical examination and diagnostic testing, communication skills, clinical reasoning and treatment planning, and clinical skills—were drafted based on Wonkwang University's competency framework and the evaluation criteria of the Institute of Korean Medicine Education & Evaluation, and the corresponding items underwent the same content validity evaluation described above. Each domain was assessed using a single item, with importance and performance rated independently on a 5-point Likert scale. Completion took approximately 15 min. Recruitment notices and an online survey link were distributed throughout the class community so that students who had completed clerkships could participate voluntarily. The survey was open from 7 to 28 November 2025.

### 2.4 Interviews

A semi-structured interview guide was developed based on a preliminary analysis of the survey results. The main questions covered following domains: overall perception of clinical clerkship education, CPX, OSCE, graduation examinations, clinical rotations, community-based clerkship, proposals for improving clerkship education, student competency, and professional growth (Supplementary File 2). Interview participants were recruited by collecting the contact details of the survey respondents who indicated that they were willing to be interviewed at the end of the survey. Snowball sampling was used to obtain a wider range of participants, including students from different clerkship groups. Interviews were conducted between 13 and 27 November 2025.

Interviews were conducted by a co-investigator (SYH) who holds a doctorate in education and is not involved in delivering the existing clerkship curriculum. Each participant took part in a single one-to-one interview, held via videoconferencing software (Zoom Workplace; Zoom Communications, San Jose, CA, USA), and lasting approximately 1 h. All interviews were recorded with the participants’ consent. Recruitment continued until saturation was reached; that is, until the data collected and analyzed were judged sufficient that further collection would add nothing new (20). One member of the research team (EC) was known to participants due to involvement in the clinical clerkship, so that member did not take part in data collection. Participants were assured that their responses would not bear on the course assessment, and the team revisited throughout the analysis how this prior relationship might have shaped what participants chose to disclose.

### 2.5 Analysis

The survey results were first summarized as response frequencies and proportions for each item. For the open-ended responses, either the number of respondents per response category or the full content of each respondent's answer was presented. Borich scores were computed as Σ[(Importance − Performance) × mean Importance]/*N*. A paired *t*-test examined the statistical significance of the difference between importance and performance for each of the five competency domains, with significance set at *p* < 0.05. All analyses were performed using GraphPad Prism (version 10.2.2; GraphPad Software, Boston, MA, USA). Priorities among learners’ educational needs were then determined using the Borich Needs Assessment model, which multiplies the difference between importance and performance by the mean importance score. Finally, the Locus for Focus model was used to visually display these priorities. In this model, competencies are distributed across four quadrants according to importance, performance discrepancy, and level of importance, with Quadrant I representing the highest-priority needs, Quadrants II and IV moderate needs, and Quadrant III the lowest (21). The two models may yield different priority orderings for a given domain because the Borich model weights the importance–performance discrepancy by mean importance score, whereas the Locus for Focus model prioritizes domains based on both the level of importance and the magnitude of discrepancy without numerical weighting.

All interviews were transcribed in full, and passages directly related to the survey items were extracted and compared with the survey results. The transcripts were also analyzed as raw data using qualitative thematic analysis in ATLAS.ti (web version; ATLAS.ti Scientific Software Development GmbH, Berlin, Germany). SYH conducted all interviews. SJ assisted with participant recruitment and interview scheduling and led the qualitative analysis, with the researcher involved in the clinical clerkship course (EC) not participating in data collection. Two researchers (SYH and SJ) independently reviewed and coded the full transcripts before the two met to compare their codes and resolve any discrepancies through discussion until consensus was reached. They were deductively assigned to three pre-established analytic categories—educational content, educational methods, and educational environment—defined by the research team to support the derivation of improvement measures. They were then coded and categorized through iterative comparison.

The survey results were used to identify the current status and priority needs of clinical clerkship. The interview results were used to explore in depth the causes of and solutions to the major problems identified in the survey, which were presented in an integrated manner in reporting the results. Quantitative survey and qualitative interview findings were integrated narratively. For each major theme, survey results were presented first, followed by interview data that elaborate on the underlying reasons and contextual factors.

## 3 Results

### 3.1 Characteristics of participants

Forty-two fourth-year Korean Medicine students completed the survey: 16 men (38.1%) and 26 women (61.9%). Their planned careers after graduation included salaried physicians (38.1%), military service [e.g., as a public health doctor (28.6%)], specialist training as an intern or resident (28.6%), self-employed physicians (2.4%), and full-time graduate studies (2.4%). Seven students participated in the interviews (5 women and 2 men) with planned careers as salaried physicians (*n* = 4), public health doctors (*n* = 2), and specialist training (*n* = 1) (Table 2).

**Table 2:** Demographic characteristics of participants.

Characteristics	Survey (*n* = 42)	Interview (*n* = 7)
	*N* (%)	*N* (%)
Gender		
Male	16 (38.1)	2 (28.6)
Female	26 (61.9)	5 (71.4)
Age		
23–25	26 (61.9)	0 (0.0)
26–30	14 (33.3)	7 (100.0)
31–35	1 (2.4)	0 (0.0)
36–40	1 (2.4)	0 (0.0)
Career plan		
Military service (e.g., public health doctor)	12 (28.6)	2 (28.6)
Specialist training (Intern, Resident)	12 (28.6)	1 (14.3)
Salaried physician	16 (38.1)	4 (57.1)
Self-employed physician	1 (2.4)	0 (0.0)
Graduate student (full-time)	1 (2.4)	0 (0.0)

### 3.2 Overall perceptions of clinical clerkship

Asked which forms of clerkship they had experienced most, respondents ranked patient observation first (*n* = 35), followed by assignments and presentations (*n* = 3) and CPX or OSCE (*n* = 2). Regarding the current clinical rotation component of the clerkship (24 weeks, 960 h), 81.0% judged it appropriate, 11.9% judged it too short, and 7.1% judged it too long. Most interview participants likewise described the duration as appropriate, although one observed, “*If we really wanted to compress it, the content could be condensed further*” (Participant 5).

Satisfaction with the current clerkship education was reported by 83.3% of respondents and dissatisfaction by 16.7%. Reasons for satisfaction included “there were many patients and appropriate interaction with the professors,” “my understanding of clinical skills improved,” and “observing in the clinical setting was beneficial because I could see the entire process of patient care, diagnosis, and treatment.” Reasons for dissatisfaction included “the time spent observing was not beneficial” and “(clerkship) time is insufficient and should be extended.” Eighteen participants (42.9%) reported that clerkship needed to change, citing (up to two reasons each) “variation in clerkship across departments” (*n* = 11), “limited opportunities for direct student participation” (*n* = 9), “unnecessary clerkship content” (*n* = 4), “insufficient knowledge and skills learned” (*n* = 4), and “insufficient overall education time” (*n* = 3).

### 3.3 Educational needs and priorities in Korean medicine clinical clerkship

Among the five competency domains addressed by clerkship, respondents rated “physical examination and diagnostic testing” highest in importance (mean 4.50) and “clinical reasoning and treatment planning” lowest (mean 4.19). For performance, “communication skills” was rated lowest (mean 3.69) and “history taking” highest (mean 4.02). Performance fell below importance in every domain, with “communication skills” showing the widest gap. Paired *t*-tests confirmed that this difference was statistically significant (*p* < 0.05) in all domains except “clinical reasoning and treatment planning.” Priorities derived from the Borich needs assessment model ranked, in descending order, “communication skills,” “clinical skills,” “physical examination and diagnostic testing,” “history taking,” and “clinical reasoning and treatment planning” (Table 3). In the Locus for Focus model, “physical examination and diagnostic testing” fell within the high-need region (Quadrant I) and “clinical skills” on the boundary between the high- and moderate-need regions. “Communication skills” and “history taking” fell within the moderate-need regions (Quadrants II and IV) and “clinical reasoning and treatment planning” fell within the low-need region (Quadrant III) (Figure 2).

**Table 3:** Borich needs assessment of Korean medicine clinical clerkship.

Competency domain	Importance (RCL)	Performance (PCL)	Importance – Performance			Borich score	Borich's priorities
	*M* (*SD*)	*M* (*SD*)	*M* (*SD*)	*t*	*p*-value		
History taking	4.48 (0.77)	4.02 (0.81)	0.45 (0.59)	4.946	<0.0001	2.02	4
physical examination and diagnostic testing	4.50 (0.83)	3.98 (0.75)	0.52 (0.92)	3.702	0.0006	2.36	3
Communication skills	4.33 (0.93)	3.69 (1.00)	0.64 (1.30)	3.197	0.0027	2.79	1
Clinical reasoning and treatment planning	4.19 (0.92)	3.81 (0.92)	0.38 (1.34)	1.839	0.0732	1.60	5
Clinical skills	4.38 (0.66)	3.81 (0.86)	0.57 (0.97)	3.832	0.0004	2.50	2

RCL, required competence level; PCL, present competence level; M, mean; SD, standard deviation.

**Figure 2:**
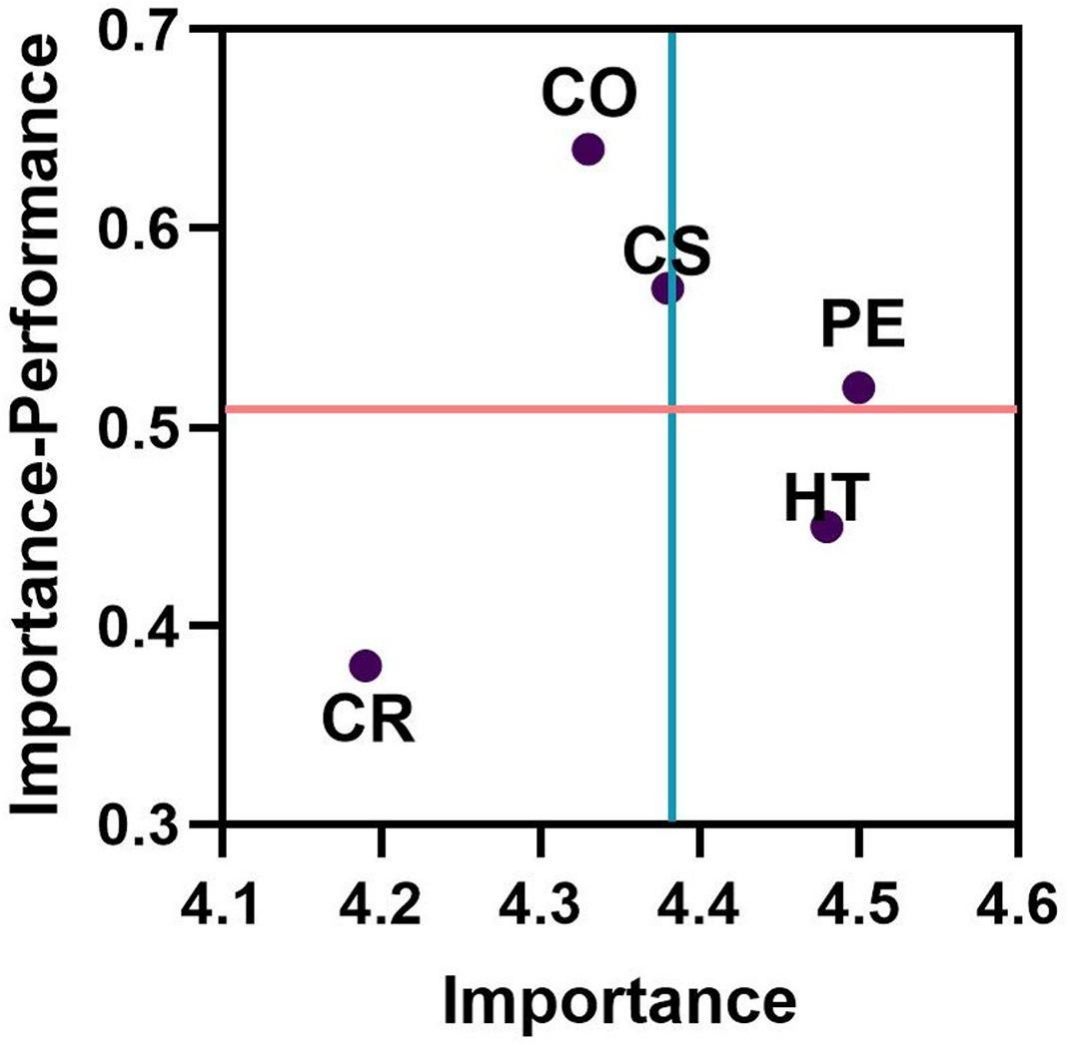
The locus for focus model for prioritizing clinical clerkship competencies. The *x*-axis represents the mean importance score and the *y*-axis the discrepancy between importance and performance (importance minus performance). The vertical line marks the grand mean of importance (4.38) and the horizontal line the grand mean of the importance–performance discrepancy (0.51). HT, history taking; PE, physical examination and diagnostic testing; CO, communication skills; CR, clinical reasoning and treatment planning; CS, clinical skills.

### 3.4 Major problems and improvement measures in clinical clerkship

#### 3.4.1 Educational content: the need to strengthen core practical training applicable to clinical practice

Learners’ needs in the “educational content” domain concerned the practical competencies and specialized knowledge expected of future Korean Medicine doctors. Regarding “physical examination and diagnostic testing”—identified as the highest-priority domain in the Locus for Focus model—interview participants indirectly mentioned the need for such training in relation to a lack of opportunities for hands-on practice, rather than a demand for more instruction in the physical examination itself. Some felt that too little class time was allocated to skills practice during clerkship and wanted more, while others valued the chance to practice skills that they had never previously attempted. “*I think it would be good to place more emphasis on skills that weren't covered in the existing curriculum or that we couldn't practice due to a lack of materials*” (Participant 5); “*We never had any class where we actually used thread embedding, acupotomy, or pharmacopuncture ourselves. But it was great to try these skills under the guidance of a resident*” (Participant 2*)*; “*I was a bit disappointed that we didn't get to directly try skills that students could perform, like cupping or needle removal, at our clerkship sites*” (Participant 2). Nevertheless, when asked whether they would be able to perform the skills they had learned during clerkship in actual practice after graduation, 40.5% responded negatively (“disagree” 35.7%; “strongly disagree” 4.8%). Interview participants similarly noted that although they had been exposed to the skills, they had few opportunities to practice them at length. “*Even if we didn't apply the skills directly to patients, it was disappointing that students weren't given as many practice opportunities as possible*” (Participant 2); “*It felt like the learning was too simplified, with the point being just that we'd tried the skill once. So I found myself wondering whether I could actually use it in real clinical practice*” (Participant 5).

On “communication skills,” which ranked first in the Borich priority, learners still reported difficulty with conversational skills as clinicians even after completing clerkship, as one comment captured: “*Communication seems to be something that gets harder the more you practice it*” (Participant 7). Several participants argued that communication training geared toward clinical settings should precede clerkships. “*I think it would help to continuously offer courses and expose students to related content for communication skills starting from the lower years*” (Participant 5); “*I think this is hard to develop through hospital clerkship*” (Participant 4); “*There's no specific (communication) training during clerkship*” (Participant 2). Others emphasized learning about clinician interactions by observing how the faculty communicated with patients during care. “*I think a clinician's demeanor and attitude can't be learned from a book—you have to watch what the professor does—so I think (communication training) should be incorporated partway through the clerkship*” (Participant 7).

Finally, some participants raised the need for training on “criteria for referral to a higher-level hospital” as a core clinical judgment skill for recognizing emergencies. As future Korean Medicine doctors who mostly provide primary care, they felt that they should learn clear criteria for judging when to transfer a patient and refer to another institution. “*Even if we don't encounter it very often, I thought we needed realistic training on when to tell a patient to go to the tertiary hospital right away*” (Participant 3).

#### 3.4.2 Educational methods: limited opportunity for active participation and for following patient care through

Regarding “educational methods,” attention was paid to how students learned during clerkships. No opportunity to participate directly in history taking or physical examination of real patients was reported by 42.9% of the survey respondents. The interviews confirmed that clerkship is primarily observation-based and participants hoped for expanded opportunities to participate in patient care at a level appropriate for students. “*I wish there were more participatory classes where students could actually try things out on patients themselves …*” (Participant 2); “*It would be better if we could see a few more patients*” (Participant 4); “*Patient cases were introduced, but we rarely encountered real patients directly*” (Participant 5).

Participants also noted that they were not given the chance to follow a patient's treatment from start to finish and that in some departments, observation itself was not possible. “*We rarely got to see initial patient consultations and examinations—it felt more like verbal explanations similar to a classroom lecture*” (Participant 5); “*In some cases, certain professors often didn't allow observation at all …*” (Participant 6). Some had no opportunity to see inpatients at all. “*We couldn't see the inpatient ward at the school hospital …*” (Participant 5). The survey pointed the same way: “not having the opportunity to see real patients in the inpatient ward” was cited as the most unsatisfactory aspect of clinical rotations.

Where interaction and feedback with faculty were concerned, question-and-answer exchanges generally proceeded smoothly; however, some participants regretted that they were given no insight into the clinical experience and judgment behind a professor's treatment choices. “*There was no explanation of the professor's clinical experience behind the choice of acupuncture treatment or prescription when treating a patient*” (Participant 3); “*In departments with patients, we had opportunities to ask the professor directly, but when there were no patients, there was nothing to ask about*” (Participant 4); “*We couldn't even get information about the patient, and we couldn't learn why a particular treatment was given*” (Participant 6).

#### 3.4.3 Educational environment: structural constraints and variation in clerkship

Turning to the “educational environment,” learners’ experiences and needs centered on the facilities and resources available for clerkship and the variation in how clerkship was run across hospitals and faculty.

In the survey, 11 respondents cited “variation in clinical clerkship across departments” as a reason why clerkship needs to change. Interview participants likewise noted that the extent of observation permitted varied by hospital and department and described variation attributable to individual faculty teaching styles. “*The scope of observation that (professors) wanted differed by department …* *In department A, there were times when we couldn't observe at all, and there were differences between hospitals and departments that weren't well communicated to us*” (Participant 1); “*There was a lot of variation across departments—I expected to see a lot of patients in practice, but it varied greatly depending on the department*” (Participant 2); “*Professors*’ *styles differ a lot—in department B we were told to study on our own, while in department C we were told to take turns observing one at a time*…*.*” (Participant 6).

Most participants described the community-based clerkship undertaken outside the university's Korean Medicine hospital setting as beneficial. They noted that university hospitals and community institutions offered distinct learning experiences and that community placement was particularly valuable because it connected directly to the career paths they realistically expected to follow. “*What I could learn at the university hospital and at a primary care institution was different, and it let me think about what would realistically be helpful for my future*” (Participant 3); “*I think I learned a lot from actually being shown how a primary care institution refers patients to a conventional medicine hospital*” (Participant 7).

Most participants were satisfied with the facilities and equipment available for clerkship, although some wanted access to a wider range of clinical equipment used in practice*.* “*We could use materials and practice as much as we wanted, so I felt fortunate to be in that kind of environment*” (Participant 5); “*It doesn't have to be expensive equipment, but I wish we had recording equipment to use for OSCE*” (Participant 5); “*I thought the equipment itself was all in good condition*” (Participant 6); “*I wish there were more varied clinical equipment and medical devices available to try out, and more rooms (for practicing skills)*” (Participant 1).

## 4 Discussion

This mixed-methods study examined the current state of clinical clerkship and learner needs among fourth-year Korean Medicine students at Wonkwang University, intending to identify directions for improving Korean Medicine clerkship. Regarding educational methods, clerkships were predominantly observation-based, limiting opportunities for active participation in patient care. In terms of contents, the need for communication and clinical skills ranked highest, whereas the perceived need for clinical reasoning was comparatively low. Concerning the environment, clerkship varied across hospitals and departments. These findings indicate a need to shift the Korean Medicine clerkship from passive observation to active participation in patient care. Priorities and detailed improvements suggested in this study can serve as a formative diagnosis for redesigning a learner-centered, competency-based clerkship model in traditional medicine.

### 4.1 Insufficient participation in actual patient care

Survey and interview data agreed that most learners at Wonkwang University's Korean Medicine clerkship did not take part in the care process—for instance, history taking or physical examination—with real patients and that clerkship remained predominantly passive observation. This can be attributed in part to the absence of a clerkship structure and standards that explicitly require direct student participation, but more fundamentally to a set of interlocking factors, including Korea's Medical Service Act, which prohibits unlicensed medical practice, and broader recent changes in the clinical environment. Article 27 of the Act explicitly prohibits medical practice by unlicensed individuals. Pre-licensure students may perform medical acts only under the direct guidance and supervision of a “supervising professor.” Examining patients without a professor present, or under the guidance of a clinical instructor or resident alone, can be problematic (22, 23). This structural constraint, combined with the hospital culture in Korea that prioritizes patient safety over educational participation, tends to make student involvement in patient care more passive (24).

Some interview participants reported that even observation was restricted for inpatients; the COVID-19 pandemic prompted stronger infection control within hospitals and fostered a culture that further restricted student access, particularly to inpatient wards, an effect that has partly persisted (25, 26). Therefore, clerkship protocols must clearly define the roles and scope within which students may progressively participate in patient care under the direct supervision of a supervising professor with patient safety as a precondition. In health professions education, EPAs offer an established means of specifying such graduated responsibilities (27) and adapting them to the Korean Medicine context could give both students and supervisors a shared guideline for what a learner may do unsupervised and when. Policy discussion is also needed to establish a clearer legal basis for student participation in patient care for educational purposes so that such participation is institutionally supported within a proper supervisory framework.

### 4.2 The need for systematic curriculum design to strengthen communication and clinical skills

“Communication skills,” which ranked first in the Borich priority analysis, showed the largest gap between importance and performance among the five competency domains. The interviews confirmed that although observing communication between faculty and patients during care was regarded as valuable for learning, no concrete communication training was provided during clerkships, leaving learners struggling with conversational skills as clinicians. Opportunities for practical communication skill development within clerkships fall short of what learners need.

Communication competency is difficult to develop through short-term training or incidental exposure during clerkship alone. Sustained improvement requires repeated learning through a longitudinal, spiral curriculum spanning the early years through the entire clerkship (28). At present, however, Wonkwang University has no course dedicated to communication instruction, and the subject is addressed indirectly only within the second-year university Medical Ethics course. Communication-related courses appear elsewhere in Korean Medicine education. Still, they remain confined to a single course at the single-year level, and a longitudinal curriculum spanning clerkship has not yet been established (29, 30). Therefore, Korean Medicine education should introduce communication-related courses systematically from the early years and redesign the curriculum with a spiral structure that allows repeated and progressively deepening learning throughout clerkships, integrated with clinical competency training. Role modeling, in which faculty members make their thought processes and communication style visible to learners in a clinical setting, can produce effective learning (31). Therefore, standardized teaching methods that enable faculty members to serve as effective communication role models should be established together with systematic faculty development (31, 32).

Clinical skills ranked second among the five competency domains at the need level, indicating that students required substantially more opportunities to learn them. In the Locus for Focus model, “clinical skills” fell on the boundary between high- and moderate-need regions, and 40.5% of respondents in the survey doubted that they would be able to perform the skills learned during clerkship in actual practice after graduation. The interviews likewise showed that although students encountered a range of skills during clerkship, they were given few opportunities to practice them and thus lacked the confidence that actual practice demands.

If skills training remains limited to experiencing a skill once, as in the current curriculum, learners will struggle to develop the competency to judge soundly and perform appropriately in actual practice (33). Prior research indicates that simulation-based medical education grounded in deliberate practice can markedly improve skill performance by providing immediate feedback and repeated training toward clearly defined performance goals (33, 34). Therefore, learning objectives should be defined explicitly against the skill competencies required in practice after graduation, and the clerkship curriculum should be designed to ensure sufficient practice time, repeated training, and formative assessment with feedback (4). A comprehensive summative assessment of clinical skills upon completion of the curriculum would then confirm that learners have achieved the intended outcomes, ensuring that they can perform these skills appropriately in practice. Embedding such assessments within a programmatic approach in which many low-stakes observations accumulate into a defensible judgment of competence (35) would align clinical skills training in Korean Medicine with contemporary CBE practice.

While “communication skills” ranked first in the Borich analysis, “physical examination and diagnostic testing” was placed in the highest-priority region (Quadrant I) of the Locus for Focus model despite ranking third in the Borich analysis. This is because “physical examination and diagnostic testing” recorded the highest mean importance score (4.50) among all five domains, but its importance–performance discrepancy was smaller than those of communication and clinical skills, resulting in a lower Borich rank. The Locus for Focus model, which considers both the level of importance and the discrepancy simultaneously without numerical weighting, identified it as the highest-priority need. This difference reflects the complementary nature of the two models, and together they suggest that “physical examination and diagnostic testing” warrants attention in curriculum redesign alongside communication and clinical skills.

### 4.3 Perceived low need for clinical reasoning training and the need for appropriate teaching strategies

In the Borich analysis, “clinical reasoning and treatment planning” recorded the lowest mean importance score (4.19) of the five domains, fell within the low-need region (Quadrant III) of the Locus for Focus model, and was the only domain for which the paired *t*-test showed no statistically significant difference between importance and performance. The interviews pointed to a possible reason: the faculty members did not sufficiently explain the clinical experience underlying their choice of acupuncture treatment or prescription.

Clinical reasoning is a core medical competency that involves the collection and integration of diverse clinical information about a patient and formulation of a diagnosis and treatment plan (36). In Korean Medicine, pattern identification, known as “byeonjeung”, is a unique diagnostic process. It involves gathering comprehensive patient information through the four diagnostic methods: inspection, listening and smelling, inquiry, and palpation. This approach determines the appropriate treatment and prescription, evaluates treatment effects, and predicts prognosis, forming the foundation for treatment decisions (37). However, clinical reasoning is often not explicitly taught in healthcare curricula (38, 39) and is instead acquired incidentally in clinical settings (40). Medical education that prioritizes memorization and test performance has also been shown to leave students with fragmented knowledge (41). Without a teaching strategy aimed specifically at clinical reasoning, competency may be difficult to develop. This may explain the low perceived need and importance that our survey recorded. A substantial proportion of learners may lack both understanding of and prior instruction in core clinical reasoning concepts (40), and so may not yet recognize what competency involves.

Clinical reasoning is not effectively improved by teaching generic thinking processes alone; it requires an instructional design calibrated to the learner's level (42). In the early stages of learning, self-explanation—where learners explain the signs and symptoms of a case in relation to pathophysiological mechanisms—proves to be effective. This is followed by deliberate reflection, which involves comparing and contrasting similar conditions. In later years, once learners have accumulated enough clinical knowledge, it is beneficial to engage in training that involves gathering and evaluating patient information in a setting that resembles actual practice (42). This suggests that curriculum design should build clinical reasoning not only through theoretical knowledge acquisition but also through repeated case-based practice and feedback in environments that closely resemble real clinical work. Shifting away from assessments that focus on memorization and instead evaluating the reasoning process itself would encourage learners to develop clinical reasoning habits and make their competencies more visible to those who currently undervalue them.

### 4.4 Variation in clinical clerkship across departments

Among the reasons respondents provided for needing change in clerkship, “variation in clerkship across departments” drew the highest response rate. The interviews likewise pointed to differences in the depth and manner of clerkship supervision from one faculty member to another, across Wonkwang University's three affiliated Korean Medicine hospitals and across departments within the same hospital, as well as differences in the length of clerkship assigned by departments. The same department was offered at more than one affiliated hospital, and educational content was sometimes not clearly divided between sites, producing redundancies or inconsistencies.

This problem has been widely reported in clerkship research, both in Korea and internationally. Variation in clerkship effectiveness arises because the elements constituting the clerkship environment—faculty supervision and patient composition—differ across departments and institutions; reducing it has been shown to require not only standardization across institutions but also faculty development to manage variation among individual faculty within an institution (43). To minimize the variation observed, educational content and roles should be clearly differentiated among hospitals offering the same department at multiple sites. Additionally, clerkship duration and core content should be standardized by department. Faculty development aimed at reducing variations in supervision should be implemented alongside these measures, with a system for sharing and coordinating clerkship operations across institutions.

### 4.5 Limitations of this study

This study is a case analysis confined to a single institution; therefore, it does not capture the clerkship environment across all 12 colleges of Korean Medicine nationwide. The mixed-methods analysis was also conducted with only a subset of learners who had completed clerkship, so the sample was small. The response rate of 46.2% (42 of 91 eligible students) also raises questions about sample representativeness. Because systematic demographic data were not collected for non-respondents, we cannot confirm whether the sample is representative of the full 4th-year class. Since interview participants volunteered, selection bias may have operated, in that more proactive learners, or those whose experiences and perceptions resembled one another, may have been more likely to take part. Additionally, transcript verification with interviewees (member checking) was not conducted, which we acknowledge as a limitation of the qualitative procedure. Moreover, data were collected at a single time point after the clerkship ended, which makes it difficult to track how learners’ needs evolve and limits any claim about causal relationships.

Against these constraints, this study is the first to apply a systematic needs assessment and mixed-methods approach to learners’ perceptions of Korean Medicine clerkship and to identify areas for improvement in educational content, methods, and environment. The results offer concrete implications for strengthening clinical skills, communication, and clinical reasoning in traditional medicine education and provide one of the few learner-derived evidence bases for CBE implementation outside the biomedical curricula in which such frameworks were developed.

These findings indicate the following steps: The clerkship curriculum at the College of Korean Medicine should be redesigned, and learners’ perceptions of the new curriculum should be investigated and compared with the present results to establish whether improvements have occurred. Future multicenter research could examine whether contextually adapted EPAs are feasible and useful for clarifying graduated student responsibilities in Korean Medicine clerkships. If similar needs are confirmed through multicenter studies, future research and policy discussions may consider developing shared learning outcomes and exploring the feasibility of standardized clerkship and assessment approaches. The potential relevance of these findings to the national licensing examination should be examined separately using broader stakeholder and national-level evidence. Ongoing research to analyze the needs of stakeholders in medical education, including traditional medicine, is important for building toward a competency-based framework. Future studies could expand the sample through nationwide multicenter work and longitudinally analyze changes in learners’ perceptions and needs, thereby strengthening the reliability and generalizability of the findings.

## 5 Conclusions

In this institutional case, learners’ accounts suggest that Korean Medicine clerkship may benefit from moving beyond predominantly observational experiences toward structured participation, repeated skills practice, and explicit teaching of clinical reasoning. Using an explanatory mixed-methods design partially overlapping data collection, this study integrated survey and interview findings and identified priority areas for improvement. The Borich analysis identified high learner needs in communication and clinical skills, while the Locus for Focus model also highlighted physical examination and diagnostic testing. Clerkship remained predominantly observation-based and delivery varied across hospitals and departments. These findings offer valuable evidence for the redesign of the Korean Medicine clerkship curriculum. The priority analysis may inform the local redesign and future evaluation of Korean Medicine clerkship. These findings may also provide preliminary learner-informed evidence for considering how competency-based approaches could be adapted to traditional medicine education.

## Data Availability

The original contributions presented in the study are included in the article/Supplementary Material, further inquiries can be directed to the corresponding author.
