# Supplementary material for: From observation to participation: a needs assessment of clinical clerkship in Korean medicine toward competency-based education
Source: Front Med (Lausanne). 2026 Oct 14;13:1969260. doi: 10.3389/fmed.2026.1969260 (PMC13714917; doi:10.3389/fmed.2026.1969260)
Supplement: Supplementary file 1 [file Supplementaryfile1.docx]

Survey items and distribution of responses

| **Survey item** | | **Response**  **N (%)** |
| --- | --- | --- |
| **Section 1. Characteristics of participants** | |  |
| What is your gender? | |  |
|  | Male | 16 (38.1) |
|  | Female | 26 (61.9) |
| What is your age? | |  |
|  | 23–25 | 26 (61.9) |
|  | 26–30 | 14 (33.3) |
|  | 31–35 | 1 (2.4) |
|  | 36–40 | 1 (2.4) |
| What career path are you planning to pursue immediately after graduation? | |  |
|  | Military service (e.g., as a public health doctor) | 12 (28.6) |
|  | Specialist training (intern or resident) | 12 (28.6) |
|  | Self-employed physician | 1 (2.4) |
|  | Salaried physician | 16 (38.1) |
|  | Graduate student (full-time) | 1 (2.4) |
|  | Public institution | 0 (0.0) |
|  | Other (please specify) | 0 (0.0) |
| **Section 2. Pre-clerkship education** | |  |
| The following are questions regarding the pre-clerkship education conducted in January 2025 (patient interviews, patient safety, infection and environmental hazard prevention and response methods). | |  |
| Did the pre-clerkship education help you learn the patient interview process? | |  |
|  | Strongly disagree | 0 (0.0) |
|  | Disagree | 2 (4.8) |
|  | Agree | 30 (71.4) |
|  | Strongly agree | 10 (23.8) |
| Did the pre-clerkship education help you understand the precautions for ensuring patient safety? | |  |
|  | Strongly disagree | 0 (0.0) |
|  | Disagree | 1 (2.4) |
|  | Agree | 25 (59.5) |
|  | Strongly agree | 16 (38.1) |
| Did the pre-clerkship education help you understand infection and environmental hazard prevention and response methods? | |  |
|  | Strongly disagree | 0 (0.0) |
|  | Disagree | 0 (0.0) |
|  | Agree | 23 (54.8) |
|  | Strongly agree | 19 (45.2) |
| **Section 3. Overall perception of clinical clerkship education** | |  |
| How do you feel about the total duration (24 weeks, 960 hours) of the clinical clerkship you experienced (clinical department rotations and clinical skills training)? | |  |
|  | Appropriate | 34 (81.0) |
|  | Too long (the duration should be shortened) | 3 (7.1) |
|  | Too short (the duration should be extended) | 5 (11.9) |
| Please indicate the total duration you consider appropriate for clinical clerkship. (numeric response, in weeks) | |  |
| Please select the types of clinical clerkship you have experienced most frequently in order. | |  |
|  | Clinical observation | 35 (83.3) |
|  | Lecture-style classes | 1 (2.4) |
|  | Assignments and presentations | 3 (7.1) |
|  | CPX/OSCE | 2 (4.8) |
|  | Participation in actual patient care | 1 (2.4) |
|  | Other (please specify) | 0 (0.0) |
| Are you satisfied with the current clinical clerkship program? | |  |
|  | Strongly disagree | 1 (2.4) |
|  | Disagree | 6 (14.3) |
|  | Agree | 31 (73.8) |
|  | Strongly agree | 4 (9.5) |
| Why did you think that? [open-ended, optional] | |  |
| Do you think that the current clinical clerkship program is effectively helping to cultivate Korean Medicine doctors with clinical competence? | |  |
|  | Strongly disagree | 0 (0.0) |
|  | Disagree | 6 (14.3) |
|  | Agree | 34 (81.0) |
|  | Strongly agree | 2 (4.8) |
| Do you think that the current clinical clerkship program is effectively helping to cultivate Korean Medicine doctors with medical ethics and moral integrity? | |  |
|  | Strongly disagree | 0 (0.0) |
|  | Disagree | 2 (4.8) |
|  | Agree | 28 (66.7) |
|  | Strongly agree | 12 (28.6) |
| Do you think that the current clinical clerkship program is effectively helping to inherit and develop traditional medicine and cultivate Korean Medicine doctors with social responsibility? | |  |
|  | Strongly disagree | 0 (0.0) |
|  | Disagree | 3 (7.1) |
|  | Agree | 31 (73.8) |
|  | Strongly agree | 8 (19.0) |
| Do you think that the skills you learned during clinical clerkship can be applied directly in the clinical setting after graduation? | |  |
|  | Strongly disagree | 2 (4.8) |
|  | Disagree | 15 (35.7) |
|  | Agree | 19 (45.2) |
|  | Strongly agree | 6 (14.3) |
| Why did you think that? | |  |
|  | Insufficient clinical clerkship time | 3 (17.6) |
|  | Insufficient self-study time | 9 (52.9) |
|  | Limitations of assessment methods | 2 (11.8) |
|  | Inadequate educational facilities | 0 (0.0) |
|  | Dissatisfaction with educational content | 3 (17.6) |
|  | Other (please specify) | 0 (0.0) |
| How would you rate your level of participation in the clinical clerkship evaluation? | |  |
|  | Very low | 0 (0.0) |
|  | Low | 4 (9.5) |
|  | High | 30 (71.4) |
|  | Very high | 8 (19.0) |
| **Section 4. CPX and OSCE** | |  |
| Please list the following items in order of highest to lowest learning burden. [respondents ranking each item first; n (%)] | |  |
|  | CPX role-play (physician–patient) | 20 (47.6) |
|  | CPX | 3 (7.1) |
|  | OSCE | 4 (9.5) |
|  | Portfolio (CPX role-play, OSCE) | 15 (35.7) |
| Which skill did you find most difficult to perform? [open-ended] | |  |
| Which skill (refer to the table) do you find most helpful? [open-ended] | |  |
| Do you think the portfolio (CPX role-play, OSCE) that records and reflects on your performance in clinical skills practice is helpful? | |  |
|  | Strongly disagree | 4 (9.5) |
|  | Disagree | 15 (35.7) |
|  | Agree | 18 (42.9) |
|  | Strongly agree | 5 (11.9) |
| Do you think the hospital CPX helped develop competencies such as medical history taking, performing physical examinations, educating patients, and establishing treatment plans in situations similar to actual clinical settings? | |  |
|  | Strongly disagree | 1 (2.4) |
|  | Disagree | 4 (9.5) |
|  | Agree | 26 (61.9) |
|  | Strongly agree | 11 (26.2) |
| Do you think CPX role-play helped develop competencies such as medical history taking, performing physical examinations, educating patients, and establishing treatment plans in situations similar to actual clinical settings? | |  |
|  | Strongly disagree | 2 (4.8) |
|  | Disagree | 4 (9.5) |
|  | Agree | 29 (69.0) |
|  | Strongly agree | 7 (16.7) |
| Please list any equipment or facilities you believe are necessary for smooth clinical skills practice. [open-ended, optional] | |  |
| **Section 5. Clinical rotations** | |  |
| What percentage of the total training period (24 weeks, 960 hours) did residents spend conducting education during clinical rotations? | |  |
|  | 0 ≤ ≤ 20% | 1 (2.4) |
|  | 20< ≤40% | 11 (26.2) |
|  | 40< ≤60% | 19 (45.2) |
|  | 60< ≤80% | 9 (21.4) |
|  | 80< ≤100% | 2 (4.8) |
| How often did you have the opportunity to directly participate in history taking or performing physical examinations on actual patients during your clinical rotations? [numeric response] | |  |
| What was the most dissatisfying aspect you experienced during your clinical rotations? | |  |
|  | Lack of attention to students | 8 (19.0) |
|  | Absence or inadequacy of orientation | 4 (9.5) |
|  | Insufficient explanation of major-specific knowledge | 7 (16.7) |
|  | Frequently changing practicum schedules | 4 (9.5) |
|  | Difficulty interacting with patients as a student (patients' reluctance) | 12 (28.6) |
|  | Other (please specify) | 7 (16.7) |
| **Section 6. Community-based clerkship** | |  |
| Please select all institutions you experienced during community-based clerkship [multiple responses allowed] | |  |
|  | Korean Medicine clinic | 41 (97.6) |
|  | General Korean Medicine hospital (excluding hospitals affiliated with colleges of Korean Medicine) | 5 (11.9) |
|  | Long-term care hospital or general hospital | 3 (7.1) |
|  | Public medical institution | 0 (0.0) |
|  | Other (please specify) | 0 (0.0) |
| How do you feel about the total duration (80 hours) of the community-based clerkship you participated in? | |  |
|  | Very short | 1 (2.4) |
|  | Short | 3 (7.1) |
|  | Appropriate | 31 (73.8) |
|  | Long | 6 (14.3) |
|  | Very long | 1 (2.4) |
| Have you ever been rejected by clinical clerkship sites due to community-based clerkship documents? | |  |
|  | Yes | 2 (4.8) |
|  | No | 40 (95.2) |
| If the community-based clerkship was helpful, please describe specifically in what ways it was beneficial. [open-ended, optional] | |  |
| If there are any areas that need improvement in the community-based clerkship, please describe them specifically. [open-ended, optional] | |  |
| **Section 7. Future improvements to clinical clerkship** | |  |
| Please rank the goals of clinical clerkship education that you consider important in order of priority. | |  |
|  | Preparation for practice as a Korean Medicine doctor | 22 (52.4) |
|  | Career exploration as a Korean Medicine doctor | 9 (21.4) |
|  | Review and application of previously acquired knowledge | 5 (11.9) |
|  | Assessing suitability for each clinical discipline | 6 (14.3) |
|  | Other (please specify) | 0 (0.0) |
| Please rate the importance of each component in the clinical clerkship curriculum as you perceive it. (1 = not important at all, 5 = very important) | |  |
|  | History taking | 4.48 ± 0.77 |
|  | Physical examination and diagnostic testing | 4.50 ± 0.83 |
|  | Communication skills (with patients and among healthcare providers) | 4.33 ± 0.93 |
|  | Clinical reasoning and treatment planning (including differential diagnosis) | 4.19 ± 0.92 |
|  | Clinical skills (procedures, emergency care, prescribing, treatment) | 4.38 ± 0.66 |
| Please rate how well you think the learning content of the clinical clerkship curriculum (each item) is actually being implemented. (1 = not at all, 5 = very much so) | |  |
|  | History taking | 4.02 ± 0.81 |
|  | Physical examination and diagnostic testing | 3.98 ± 0.75 |
|  | Communication skills (with patients and among healthcare providers) | 3.69 ± 1.00 |
|  | Clinical reasoning and treatment planning (including differential diagnosis) | 3.81 ± 0.92 |
|  | Clinical skills (procedures, emergency care, prescribing, treatment) | 3.81 ± 0.86 |
| Do you think the current clinical clerkship program should be changed? | |  |
|  | Strongly disagree | 0 (0.0) |
|  | Disagree | 24 (57.1) |
|  | Agree | 17 (40.5) |
|  | Strongly agree | 1 (2.4) |
| Why do you think the clinical clerkship program at colleges of Korean Medicine needs to change? [up to two responses] | |  |
|  | Failure to follow the clinical clerkship plan | 0 (0.0) |
|  | Insufficient overall education time | 3 (16.7) |
|  | Variation in clinical clerkship across departments | 11 (61.1) |
|  | Insufficient knowledge and skills learned | 4 (22.2) |
|  | Difficult-to-understand specialized knowledge | 0 (0.0) |
|  | Unnecessary clerkship content | 4 (22.2) |
|  | Limited opportunities for direct student participation | 9 (50.0) |
|  | Other (please specify) | 0 (0.0) |
| Do you believe the current evaluation methods for clinical clerkship should be changed? | |  |
|  | Strongly disagree | 2 (4.8) |
|  | Disagree | 30 (71.4) |
|  | Agree | 9 (21.4) |
|  | Strongly agree | 1 (2.4) |
| Why do you think the current evaluation methods for clinical clerkship education at colleges of Korean Medicine need to change? [up to two responses] | |  |
|  | Evaluation criteria are not clearly presented | 3 (30.0) |
|  | Evaluation methods are not valid | 5 (50.0) |
|  | Feedback on evaluation results is insufficient | 1 (10.0) |
|  | Does not sufficiently reflect overall clinical competency | 6 (60.0) |
|  | Other (please specify) | 0 (0.0) |
| In what ways do you think the clinical clerkship guidelines were helpful for your clinical clerkship? [multiple responses allowed] | |  |
|  | Clinical observation | 19 (45.2) |
|  | Lecture-style classes | 8 (19.0) |
|  | Assignments and presentations | 21 (50.0) |
|  | CPX/OSCE | 15 (35.7) |
|  | Participation in actual patient care | 3 (7.1) |
|  | Other (please specify) | 1 (2.4) |
| Please list the aspects of clinical clerkship management that you feel most need improvement. [open-ended, optional] | |  |

Values are n (%) unless otherwise indicated; importance and performance ratings are mean ± standard deviation. Percentages are calculated on the full sample (N = 42) except where an item was answered only by a subgroup: reasons why clinical skills were judged not directly applicable (n = 17), reasons why the clerkship program needs to change (n = 18), and reasons why the assessment methods need to change (n = 10). For items marked as allowing multiple responses, percentages sum to more than 100. Items marked [open-ended] or [numeric response] were free-text and are summarized in the main text. CPX, clinical performance examination; OSCE, objective structured clinical examination.
