# Supplementary material for: From observation to participation: a needs assessment of clinical clerkship in Korean medicine toward competency-based education
Source: Front Med (Lausanne). 2026 Oct 14;13:1969260. doi: 10.3389/fmed.2026.1969260 (PMC13714917; doi:10.3389/fmed.2026.1969260)
Supplement: Supplementary file 2 [file Supplementaryfile2.docx]

Interview guide

**Overall Perception of Clinical Clerkship Education**

- What was the most memorable aspect of your 7-month clinical clerkship?
- Were there any differences between your expectations before starting the clinical clerkship and the reality after completing it?
- How do you feel about the total duration (24 weeks, 960 hours) of the clinical clerkship you experienced (clinical department rotations and clinical skills training)?
- Was there effective interaction between professors and students throughout the clinical clerkship?
- Is there sufficient integration between pre-clinical education and actual clinical clerkship?
- Do your think the facilities and equipment provided for clinical clerkship are adequate?

**CPX / OSCE / Graduation Exams**

- What improvements would you suggest for the CPX, OSCE and graduation exams?
- What are the primary differences between CPX role-playing and hospital CPX?
- What are the respective advantages and disadvantages of CPX role-playing and hospital CPX?
- Which components of the OSCE did you find most useful, and which were the most challenging?

**Clinical Rotation**

- What were the perceived strengths and limitations during your clinical rotations in each department?
- How are students involved in direct patient care during clinical rotation?
- What proportion of clinical clerkship time is facilitated by residents?
- What was your evaluation of the one-week rotation at a long-term care hospital during clinical clerkship at Jeonju Korean Medicine Hospital?

**Primary Care Clerkship**

- At which institution did you complete your primary care clerkship?
- What were your key observations and reflections during your primary care clerkship?
- Were there any administrative or logistical difficulties encountered during the preparation for the primary care clerkship
- What are your intended career paths following graduation?
- How are the current clinical clerkship guidelines being utilized in your clinical clerkship?

**Proposals for Improvement in Clinical Clerkship Education**

- What measures could be implemented to encourage more proactive student participation in patient care during clinical training?
- Do you have any suggestions regarding activities or curriculum for the upcoming extension of clinical clerkship hours(to 300 hours)?
- In what ways should the clinical clerkship guidelines be improved?
- How are the current clinical clerkship guidelines being utilized in your practice?

**Student Competency and Professional Growth**

- In what area have you experienced the most significant professional growth through your clinical clerkship?
- Which clinical competencies do you feel need further development in the future?
- What essential elements must be learned during clinical clerkship to become a competent primary care physician and a Korean Medicine doctor?
- Given the individual variation in communication skills, what type of educational intervention is necessary to enhance patient-physician communication?

These are all the questions we have. If you have any other comments, please feel free to tell us. Thank you for your participation.
